# Melatonin Activates AMPK Pathway to Regulate the Regeneration of Slow Muscle Fibers in Skeletal Muscle Injury Repair

**DOI:** 10.1096/fj.202501549RR

**Published:** 2025-10-12

**Authors:** Wei Xie, Zhengchao Hong, Yuxin Xie, Kangyan Hou, Songhan Li, Mingxin Fang, Yupeng Guan, Zhixuan Zhang, Miao Zhang

**Affiliations:** ^1^ Department of General Medicine First Affiliated Hospital of Gannan Medical University Ganzhou China; ^2^ Scientific Research Center The Seventh Affiliated Hospital of Sun Yat‐Sen University Shenzhen Guangdong China; ^3^ Department of Pathology The First Affiliated Hospital of Gannan Medical University Ganzhou China; ^4^ Department of Preventive Medicine Gannan Medical University Ganzhou China; ^5^ Sun Yat‐Sen University School of Medicine Sun Yat‐Sen University Shenzhen China; ^6^ Department of Physical Education Sun Yat‐Sen University Guangzhou China

**Keywords:** AMPK, melatonin, mitochondria, skeletal muscle injury repair, slow muscle fibers

## Abstract

The process of skeletal muscle regeneration entails alterations in the relative composition of muscle fiber types, yet the regulatory mechanisms remain incompletely understood. This study aimed to investigate the role of melatonin in regulating the regeneration of slow muscle fibers during skeletal muscle repair and its underlying mechanisms. Using a tibialis anterior muscle frostbite model in 6–8‐week‐old male C57BL/6J mice, in vivo experiments revealed that intraperitoneal melatonin administration significantly increased Myh7/Myh2 protein expression while reducing Myh1/Myh4 levels. In vitro, melatonin‐treated C2C12 myoblasts exhibited elevated oxygen consumption, mitochondrial mass, mitochondrial respiratory chain complexes activity, ATP production, mtDNA content, and membrane potential, alongside reduced LDH activity and ROS levels. Transcriptional upregulation of genes linked to mitochondrial complexes assembly, oxidative phosphorylation, and ATP synthesis was observed. Mechanistically, melatonin activated the AMPK/PGC‐1α pathway, as evidenced by Compound C (AMPK inhibitor) pretreatment reversing these effects, decreasing p‐AMPK/AMPK ratios, PGC‐1α, and slow fiber markers (Myh7/Myh2), while increasing ROS and fast fiber marker (Myh4). The results indicate that melatonin facilitates the formation of slow‐twitch fibers during muscle repair by augmenting mitochondrial function through the AMPK/PGC‐1α signaling pathway. Consequently, these findings imply that melatonin improves mitochondrial function via AMPK/PGC‐1α signaling, thereby promoting the regeneration of slow muscle fibers and facilitating the repair of skeletal muscle damage.

## Graphical abstract

The figure illustrates in vitro (melatonin‐treated C2C12 myoblasts) and in vivo (mouse frostbite‐induced tibialis anterior muscle injury with melatonin administration) models. Melatonin activates AMPK, which phosphorylates PGC‐1α to enhance mitochondrial bioenergetics (decreased ROS and LDH, increased ATP, mtDNA, membrane potential, and oxygen consumption), promoting slow‐twitch fiber differentiation (Myh7/Myh2 upregulation), suppressing fast‐twitch fibers (Myh1/Myh4 downregulation), and favoring slow‐twitch fiber type during skeletal muscle repair and regeneration. Compound C, an AMPK inhibitor, blocks this cascade.

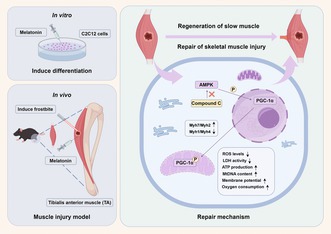

## 1 Introduction

The musculoskeletal system comprises bones, joints, and soft tissues, with the latter encompassing skeletal muscles, tendons, ligaments, and fascia, among others. Skeletal muscle represents a critical component of soft tissue, consisting of multinucleated contractile muscle cells (muscle fibers), an extracellular matrix abundant in laminin and collagen, diverse cell populations, blood vessels, and nerves. Following injury, skeletal muscle exhibits a highly coordinated response involving degeneration and regeneration processes at the tissue, cellular, and molecular levels [1]. Therefore, it is of great significance to deeply analyze the pathological process of skeletal muscle injury repair and explore effective clinical intervention methods.

Satellite cells constitute a distinct population of adult stem cells within skeletal muscle, residing beneath the basal lamina of muscle fibers and adjacent to the plasma membrane [2]. Typically, these cells remain in a quiescent state. Quiescent satellite cells are characterized by the expression of Pax7 and Myf5, and they notably overexpress signaling pathways such as AMPK, TNF‐NF‐κB, JAK‐STAT3, and Notch. In contrast, the regulatory factors involved in the cell cycle and the transcription factors pertinent to the myogenic lineage are either silent or expressed at minimal levels [3, 4]. Upon severe muscle tissue damage, caused by traumatic injuries including extensive physical activities like resistance training or exposure to myotoxins or genetic defects such as muscular dystrophy, satellite cells become activated. This activation leads to the generation of additional satellite cells and satellite‐directed progenitor cells through asymmetric division. The former replenishes the stem cell pool via self‐renewal, while the latter proliferates and differentiates into myoblasts. These myoblasts subsequently fuse to form multinucleated myotubes, establish neuromuscular junctions, and ultimately facilitate muscle regeneration [1, 5].

Myosin heavy chain (MyhC) subtypes serve as the primary classification criteria for muscle fibers. Human limb muscles comprise three MyhC subtypes: type I, type IIa, and type IIx. In contrast, rodent muscles also include type IIb fibers [6, 7]. Type I fibers, also referred to as slow‐twitch fibers, predominantly engage in oxidative metabolism, rendering them suitable for endurance activities. Conversely, type IIb and type IIx fibers, known as fast‐twitch fibers, primarily rely on glycolytic metabolism, making them ideal for short‐duration strength training. Type IIa fibers represent an intermediate category, exhibiting a mixed metabolic profile that allows for the generation of high power at relatively rapid speeds while maintaining substantial endurance [6, 8]. Given the role of hormones in influencing the plasticity of muscle fiber types, investigating the potential of melatonin to facilitate the regeneration of slow muscle fiber post‐injury may offer a promising therapeutic strategy for muscle injury repair.

Melatonin, an indoleamine predominantly secreted by the pineal gland in mammals, is synthesized from tryptophan through a series of derivatization reactions [9, 10]. Its secretion is intricately linked to light exposure; light information is conveyed to the optic chiasm via the retina and optic nerve, resulting in the inhibition of melatonin secretion. Conversely, during nighttime, when light diminishes, melatonin secretion levels increase [11]. Melatonin is widely recognized as a dietary supplement for the regulation of sleep and the resynchronization of circadian rhythm disorders. Additionally, it offers numerous other benefits, including involvement in mitochondrial homeostasis, genomic regulation, and the modulation of inflammation and immune cytokines, thereby exerting direct effects on systemic and acute anti‐inflammatory properties [12]. Melatonin is also noted for its safety profile, with no clinically established lethal dose. However, certain pharmacokinetic challenges, such as limited oral bioavailability and a short half‐life, constrain its tissue availability [13]. Recent studies have demonstrated that dietary supplementation with compounds such as arginine [14], leucine [15], and thyroid hormones [16] is implicated in the redistribution of skeletal muscle fiber types through the regulation of MyhC gene expression. Concurrently, numerous studies have shown that melatonin exerts a protective effect on tissue homeostasis and cellular function during the repair of injured skeletal muscle [17]. Consequently, this study aims to investigate the impact of melatonin on the regeneration of slow muscle fibers during the muscle injury repair process at both the tissue and cellular levels, as well as to explore its potential regulatory mechanisms.

## 2 Materials and Methods

### 2.1 Animals

Healthy male C57BL/6J wild‐type mice, aged 6 to 8 weeks, were procured from the Shanghai Model Organisms Center and maintained in a specific pathogen‐free (SPF) grade animal facility. The environmental conditions were controlled at a temperature range of 20°C to 25°C and a humidity level of 40 to 70%, with a 12‐h light/dark cycle. The mice had unrestricted access to food and water. All experimental procedures involving animals received approval from the Institutional Animal Care and Use Committee of Shenzhen Topgene Biotechnology Co. Ltd. (approval number: TOPGM‐IACUC‐2024‐0057).

### 2.2 Skeletal Muscle Injury Model

Following a 1‐week acclimatization period, anesthesia in C57BL/6J mice was induced using a small animal gas anesthesia system with 3% isoflurane. Upon achieving unconsciousness, the mice were placed on a 37°C heating pad and maintained under anesthesia via a face mask with continuous inhalation of 1% isoflurane, at an oxygen flow rate of 1 L/min. The respiratory rate was closely monitored to adjust the depth of anesthesia as necessary. Once a stable anesthetic state was confirmed, the right tibialis anterior muscle was exposed. A metal probe, cooled by liquid nitrogen and measuring 4 mm in diameter, was applied to the muscle surface for 10 s to induce cryogenic injury [18, 19]. Three days post‐injury, confirming successful modeling, subsequent experiments were conducted. Ten mice were randomly assigned to two groups (*n* = 5): the FG group, which received an injection of 5% ethanol‐saline, and the FM group, which was administered 10 mg/kg of melatonin. The Skeletal Muscle Injury protocol was developed by Miao Zhang (expert in skeletal muscle injury repair) and reviewed by two independent experts in muscle physiology. Key parameters were harmonized across all experimental groups.

### 2.3 Melatonin Administration

The dissolution of melatonin adhered strictly to the established protocol of “initial solubilization with absolute ethanol followed by dilution with physiological saline” [20]. Specifically, the procedure involved weighing the appropriate dose of melatonin, adding it to absolute ethanol, and stirring thoroughly until complete dissolution was achieved. This approach leverages melatonin's higher solubility in absolute ethanol to prevent precipitation. Subsequently, the solution was gradually diluted with physiological saline, ensuring that the ethanol volume fraction in the final solution remained stable at 5%. In relation to the ethanol content, the concentration of the melatonin solution was standardized to 1 mg/mL [21]. For mice with a body weight ranging from 20 to 25 g, using 20 g as a reference, a dosage of 10 mg/kg [22] necessitates the administration of 0.2 mg of melatonin per mouse. This corresponds to a dosage volume of 0.2 mL, which includes 0.01 mL of pure ethanol. The ethanol volume was calculated proportionally based on the required volume of the final solution, maintaining ethanol at 5% of the total solution volume, with the remainder being physiological saline. The control group (FG group) received a 5% ethanol‐saline solution prepared in the same proportion, but without melatonin, to ensure identical solvent conditions across both experimental groups. All mice were maintained in a controlled environment with a 12‐h light/12‐h dark cycle (light from 06:00 to 18:00), with treatments administered at 18:00 daily, 1 h prior to the onset of the dark phase [23], for a duration of 15 days [24]. Twenty‐four hours following the final administration [25], the mice were anesthetized using 3% isoflurane and euthanized via cervical dislocation.

### 2.4 Euthanasia

The procedure was executed as follows: Mice were initially anesthetized using a small animal gas anesthesia system with 3% isoflurane at an oxygen flow rate of 1 L/min. Anesthesia was confirmed by the absence of the corneal reflex and spontaneous limb movement, indicating complete loss of consciousness. Thereafter, 1% isoflurane was maintained to ensure a pain‐free state. Euthanasia was then performed via the cervical dislocation method under continuous anesthesia. This involved securing the head with the thumb and index finger while the tail was held with the opposite hand, followed by a swift backward pull to dislocate and fracture the cervical vertebrae, thereby instantaneously terminating central nervous function. Following the euthanasia procedure, the immediate observation of the absence of thoracic movement (indicative of respiratory cessation), the lack of cardiac pulsation (indicative of cardiac arrest), and the dilation of pupils accompanied by the loss of the pupillary light reflex confirmed the cessation of vital signs. Subsequently, sample collection was conducted.

### 2.5 Cell Culture and Grouping

C2C12 cells were maintained in Dulbecco's Modified Eagle Medium (DMEM) supplemented with 10% fetal bovine serum and 1% penicillin/streptomycin. The cultures were incubated at 37°C with 5% CO₂ and a humidity level of ≥ 95%. Initially, the cells exhibited an elliptical shape with uniform morphology and strong refractive properties, remaining suspended in the medium. After 48 h, the cells adhered to the substrate and began proliferating. When a reduction in refractive index was observed and myotube formation was absent, the medium was replaced every 2 days. Upon reaching a cell density of 80%–90%, the cultures were treated with 0.25% trypsin for either passaging or differentiation experiments. For differentiation, the cells were cultured in DMEM supplemented with 2% horse serum and 1% penicillin/streptomycin, with the medium being refreshed daily. Following 48 h of culture in horse serum, significant alterations in cell morphology were observed. The cells progressively transitioned from a spindle‐shaped to a square configuration, accompanied by an increase in cell volume and elongation of the transverse diameter, ultimately forming a distinct muscular tube structure. Additionally, the cells exhibited multinucleated characteristics, arranged in a beaded pattern. The C2C12 cells were systematically allocated into three groups: the control group (EG), the melatonin group (MG), and the melatonin plus Compound C group (MC), where Compound C serves as a potent and reversible inhibitor of AMPK by competing with ATP. Inhibition assays utilizing Compound C were performed to illustrate that melatonin modulates the regeneration of slow muscle fibers in skeletal muscle through activation of the AMPK pathway. The EG group was cultured in differentiation medium for 1 day, followed by treatment with 1 μM ethanol for 3 days. The MG group underwent culture in differentiation medium for 1 day, succeeded by treatment with 1 μM melatonin for 3 days. The MC group was cultured in differentiation medium for 1 day, pretreated with 50 μM Compound C for 1 h, and subsequently treated with 1 μM melatonin for 3 days. To ensure statistical reliability and reproducibility of the experimental outcomes, our sample size design is structured as follows: Each treatment group, including the control group, comprises three independent biological replicates, with each replicate corresponding to a distinct cell culture batch. Within each biological replicate, three technical replicates (i.e., three replicate wells) are implemented to mitigate inherent variability in the experimental procedures. Consequently, the dataset for each group in the statistical analysis consists of nine data points (3 biological replicates × 3 technical replicates). This design adheres to standard conventions in cell biology research and effectively verifies the stability and reliability of the experimental effects.

### 2.6 Tissue Collection and Processing

Following euthanasia, the tibialis anterior (TA) muscles were excised. The tissue sections were prepared utilizing the fresh frozen muscle technique. Specifically, fresh muscle tissue was embedded in OCT compound, rapidly frozen in liquid nitrogen for 1 min, and subsequently stored at −80°C. During the sectioning process, the cryostat chamber was maintained at a temperature range of −20 to −22°C. Following a 30‐min equilibration period, cross‐sectional sections with a thickness of 10 μm were cut and mounted onto polylysine‐coated slides, which were then stored at −20°C.

### 2.7 ATPase Staining

Muscle fiber typing (types I and II) was conducted utilizing the GENMED ATPase Staining Kit (Shanghai Genmed Pharmaceutical Technology, GMS80063.1). The tissue sections were subjected to alkaline activation, followed by incubation in a reaction buffer at 37°C for 30 min, and subsequently treated with activation and clearing solutions in sequence. The stained sections were then examined using a light microscope. Microscopic examination was conducted using the CellSens Standard software. ATP enzyme‐stained sections were observed in bright‐field mode within a darkroom to minimize stray light interference. A series of objectives (4×, 10×, 20×, and 40×) were employed, with a fixed exposure time of 150 ms.

### 2.8 Western Blot

Muscle tissue was homogenized on ice utilizing RIPA lysis buffer (P0013B, Beyotime, Shanghai, China) and subsequently centrifuged at 4°C to isolate the supernatant. The total protein concentration was quantified using the BCA protein quantification kit (P0010, Beyotime, Shanghai, China) and adjusted to a concentration of 2 μg/μL. Subsequently, 20 μg of total protein was loaded into each well of an SDS‐PAGE gel. Protein transfer to a 0.45 μm nitrocellulose membrane was conducted via the wet transfer method, employing a constant voltage of 100 V at 4°C for 90 min. Transfer integrity was assessed using a pre‐stained protein marker, and the uniformity of total protein bands was verified through brief staining with Coomassie blue. After washing with TBST buffer, the membrane was blocked with 5% skimmed milk for 1 h [26]. The membranes were then probed with specific primary antibodies, including MyhC1 (Proteintech, 22 282–1‐AP, dilution 1:1000), MyhC2 (Proteintech, 55 069–1‐AP, dilution 1:1000), MyhC4 (Proteintech, 20 140–1‐AP, dilution 1:1000), MyhC7 (Proteintech, 22 280–1‐AP, dilution 1:1000), AMPK (Proteintech, 10 929–2‐AP, dilution 1:1000), phosphorylated AMPK (p‐AMPK, Abcam, ab133448, dilution 1:1000), PGC‐1α (Abcam, ab191838, dilution 1:1000), and β‐actin (Proteintech, 66 009–1‐Ig, dilution 1:1000). Detection was facilitated using horseradish peroxidase (HRP)‐conjugated secondary antibodies (Abcam, ab205719/ab6721, dilution 1:5000). Image acquisition was performed using the ChemiDocTM imaging system (Bio‐Rad, Hercules, CA, USA). Quantification of band optical density was conducted utilizing ImageJ software. This involved subtracting the background using a rolling ball radius of 50 pixels from the grayscale image, selecting the band area, and measuring it three times to obtain an average value [27]. The expression level of the target protein was normalized and corrected based on the signal of the reference protein.

### 2.9 Mitochondrial Function Assays

The following methodologies were employed in this study: (1) Oxygen Consumption Rate (OCR) was determined using a Seahorse XF Analyzer. (2) ATP levels were quantified utilizing an ATP Assay Kit (Beyotime, S0027). (3) Enzyme activities, specifically those of LDH, SDH, and MDH, were evaluated using commercial kits from the Nanjing Jiancheng Bioengineering Institute. (4) Activities of the respiratory chain complexes, including Complex I (AC10158), Complex II (AC10565), and Complex IV (AC10216), were measured with kits provided by Shanghai Jizhi Biotech. (5) The mitochondrial mass in C2C12 cells was evaluated through Mito‐Tracker staining (Thermo Fisher). The analysis utilized 488 nm excitation light and a 525 ± 25 nm emission filter, with exposure times ranging from 200 to 300 ms, adjusted based on fluorescence intensity. DAPI nuclear staining was conducted using 358 nm excitation light and a 461 nm emission filter, with a fixed exposure time of 100 ms. Imaging was performed using a Zeiss 880 laser scanning confocal microscope equipped with Airyscan technology (ZEISS). Quantitative fluorescence analysis was carried out using ImageJ software. (6) Mitochondrial DNA (mtDNA) content was quantified by extracting total RNA with TRIzol (Thermo Fisher, 1 596 018), performing reverse transcription (AbmGood, G485), and conducting RT‐qPCR using the 2^−ΔΔCT^ method. (7) Flow cytometry was utilized to analyze membrane potential with TMRE (Beyotime, C2001S), mitochondrial reactive oxygen species (mtROS) with MitoSOX (Thermo Fisher, M36005), and total reactive oxygen species (ROS) with CellROX (Thermo Fisher, C10422).

### 2.10 Statistical Analysis

Data are presented as the mean ± standard deviation (SD). Prior to the application of parametric statistical analyses, the normality of the data was assessed using the Shapiro–Wilk test. The results indicated that all datasets adhered to a normal distribution (*p* > 0.05), thereby justifying the use of parametric testing methods. Comparative analyses between groups were conducted utilizing one‐way analysis of variance (ANOVA) or unpaired *t*‐tests, employing GraphPad Prism version 9. Statistical significance was determined at thresholds of *p* < 0.05, *p* < 0.01, and *p* < 0.001.

## 3 Result

### 3.1 Melatonin Influences Changes in the Relative Composition of Muscle Fiber Types Within the Injured Anterior Tibial Muscle

**FIGURE 1:**
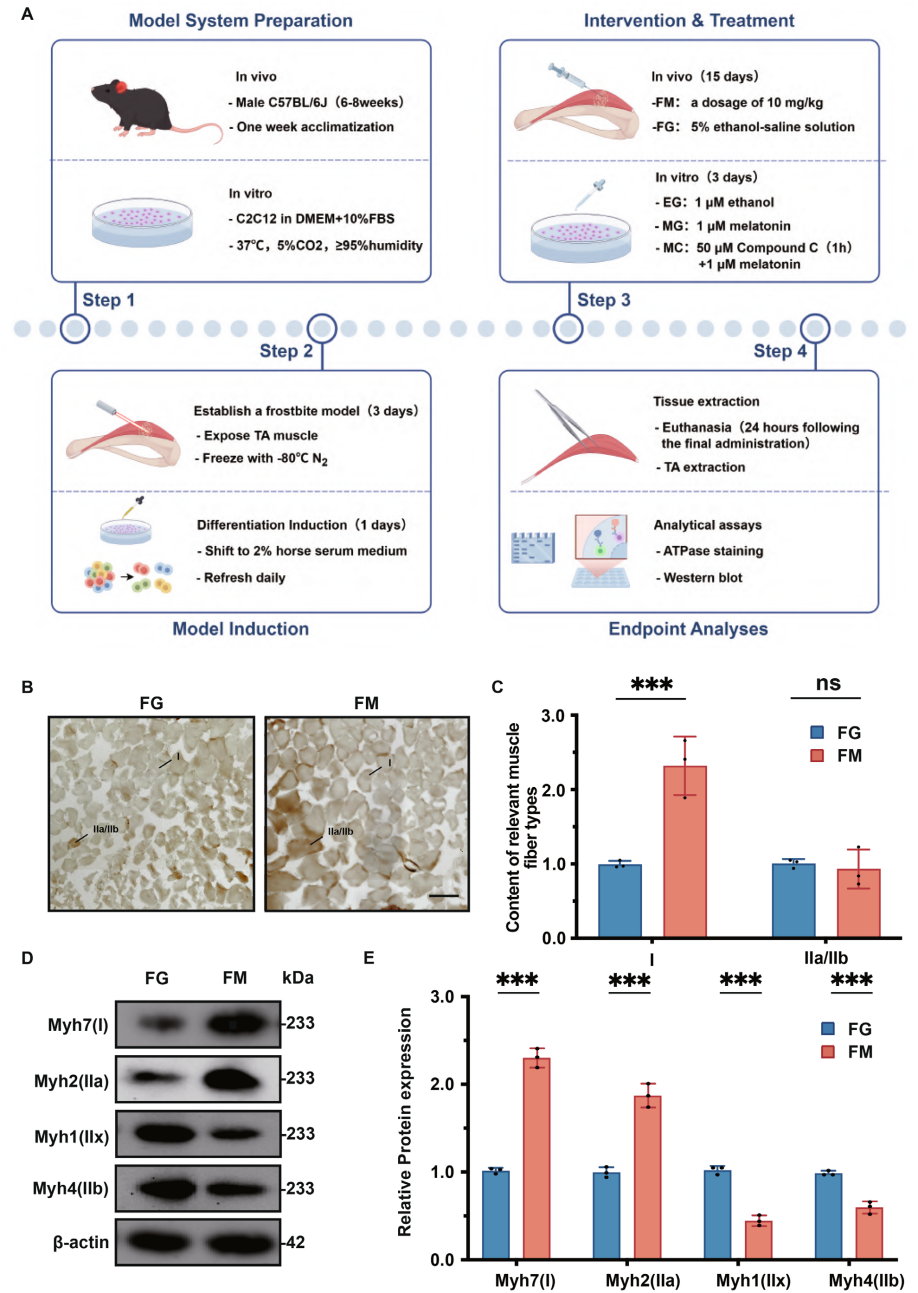
Melatonin Influences Changes in the Relative Composition of Muscle Fiber Types Within the Injured Anterior Tibial Muscle. Scale bar = 100 μm. (A) Schematic diagram of the experimental design. (B) ATPase staining was used to analyze the changes in the number of type I and type IIa/IIb muscle fibers in the FG group and the FM group, and the scale length was 100 μm. (C) The statistical analysis chart of (B). (D) Western Blot was used to analyze the changes in the protein expression levels of Myh7, Myh2, Myh1, and Myh4 in the FG group and the FM group. (E) Statistical analysis chart of (E). ***p < 0.001, compared with group FG.

Upon ATPase alkaline staining, type I muscle fibers appear thinner and light brown, whereas type II muscle fibers are relatively thicker and dark brown. The specific myosins associated with type I, type IIa, type IIx, and type IIb fibers are MyhH7, Myh2, Myh1, and Myh4, respectively. As illustrated in Figure 1, a significant increase in the number of type I muscle fibers was observed in the FM group compared to the FG group (*p* < 0.001). There was no statistically significant difference in the number of type IIa and type IIb muscle fibers between the groups. Additionally, the expression levels of Myh7 and Myh2 proteins were significantly elevated in the FM group (*p* < 0.001), while the expression levels of Myh1 and Myh4 proteins were significantly reduced (*p* < 0.001).

### 3.2 Melatonin Influences the Differentiation Propensity of C2C12 Myoblasts In Vitro

**FIGURE 2:**
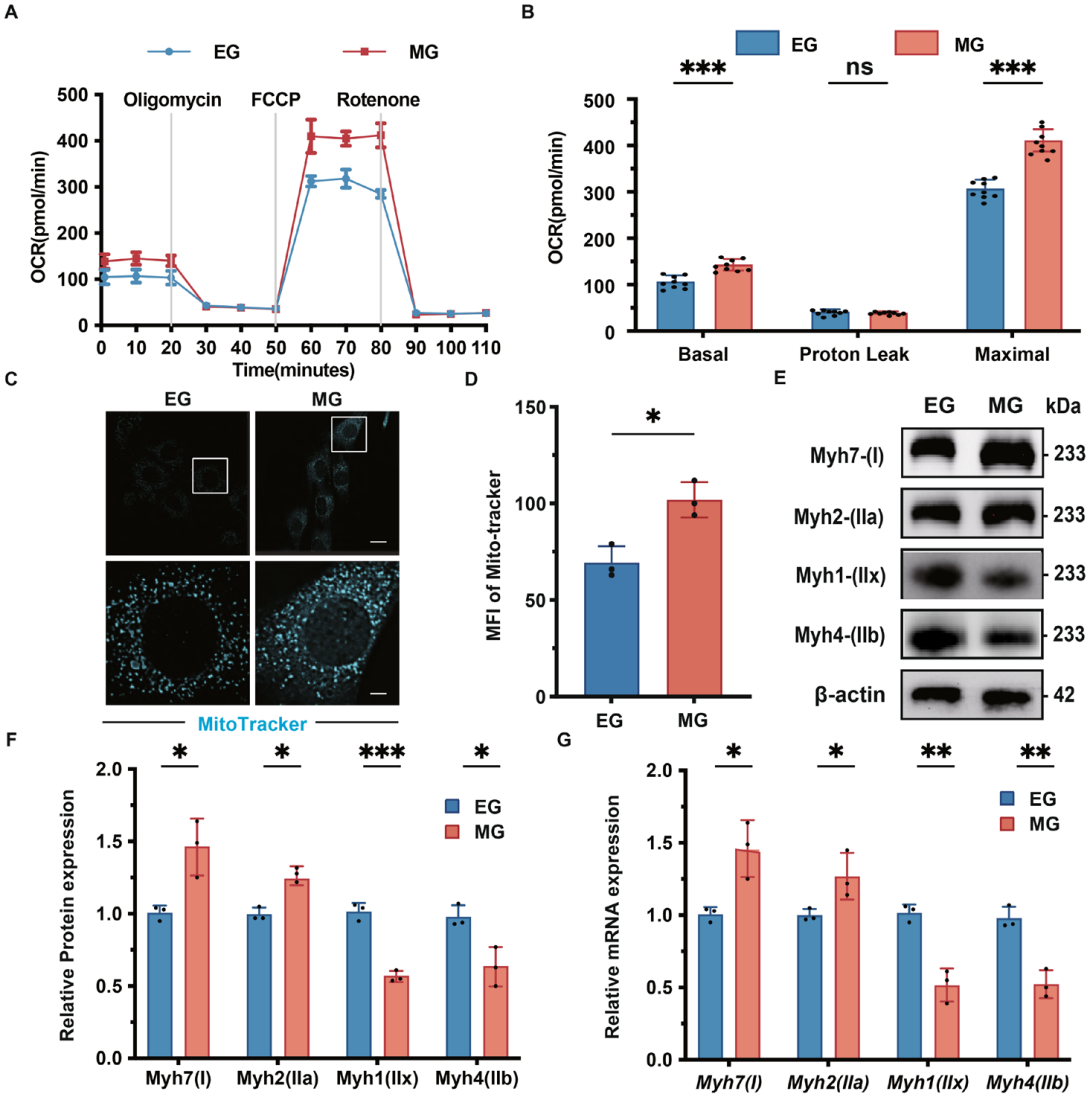
Melatonin influences the differentiation propensity of C2C12 myoblasts in vitro. C2C12 cells were cultured in the differentiation medium for 1 day and then treated with 1 μM melatonin for 3 days. (A) Changes in the OCR curve of C2C12 cells after the addition of melatonin, oligomycin, FCCP, and rotenone. (B) The statistical analysis chart of (A). (C) Immunofluorescence was used to analyze the Mito‐Tracker‐labeled mitochondria in the EG group and the MG group. Scale bar (upper) = 10 μm. Scale bar (lower) = 4 μm. (D) Statistical analysis chart of (C). (E) Western Blot was used to analyze the changes in the protein expression levels of Myh7, Myh2, Myh1, and Myh4 in the EG group and the MG group. (F) Statistical analysis chart of (E). (G) Statistical analysis graph of changes in the expression levels of Myh7, Myh2, Myh1, and Myh4 mRNA in the EG group and the MG group analyzed by real‐time quantitative PCR. *p < 0.05, **p < 0.01, ***p < 0.001, compared with the EG group.

Oxygen Consumption Rate (OCR) denotes the rate at which cells utilize oxygen. As illustrated in Figure 2A,B, the MG group exhibited a significant increase in overall oxygen consumption rate, basal respiratory rate, and maximum respiratory rate compared with the EG group (*p* < 0.001), while changes in proton leakage were not statistically significant. Figure 2C,D demonstrates that the average mitochondrial fluorescence intensity was significantly elevated in the MG group relative to the EG group (*p* < 0.05). Furthermore, as depicted in Figure 2E through 2G, the MG group showed a significant upregulation in the protein expression levels of Myh7 and Myh2 (*p* < 0.001) and mRNA transcription levels (*p* < 0.05) compared with the EG group. Conversely, there was a significant reduction in the protein expression levels of Myh1 (*p* < 0.001) and Myh4 (*p* < 0.05), as well as in mRNA transcription levels (*p* < 0.01).

### 3.3 Melatonin Enhances the Mitochondrial Function of Muscle Fibers

**FIGURE 3:**
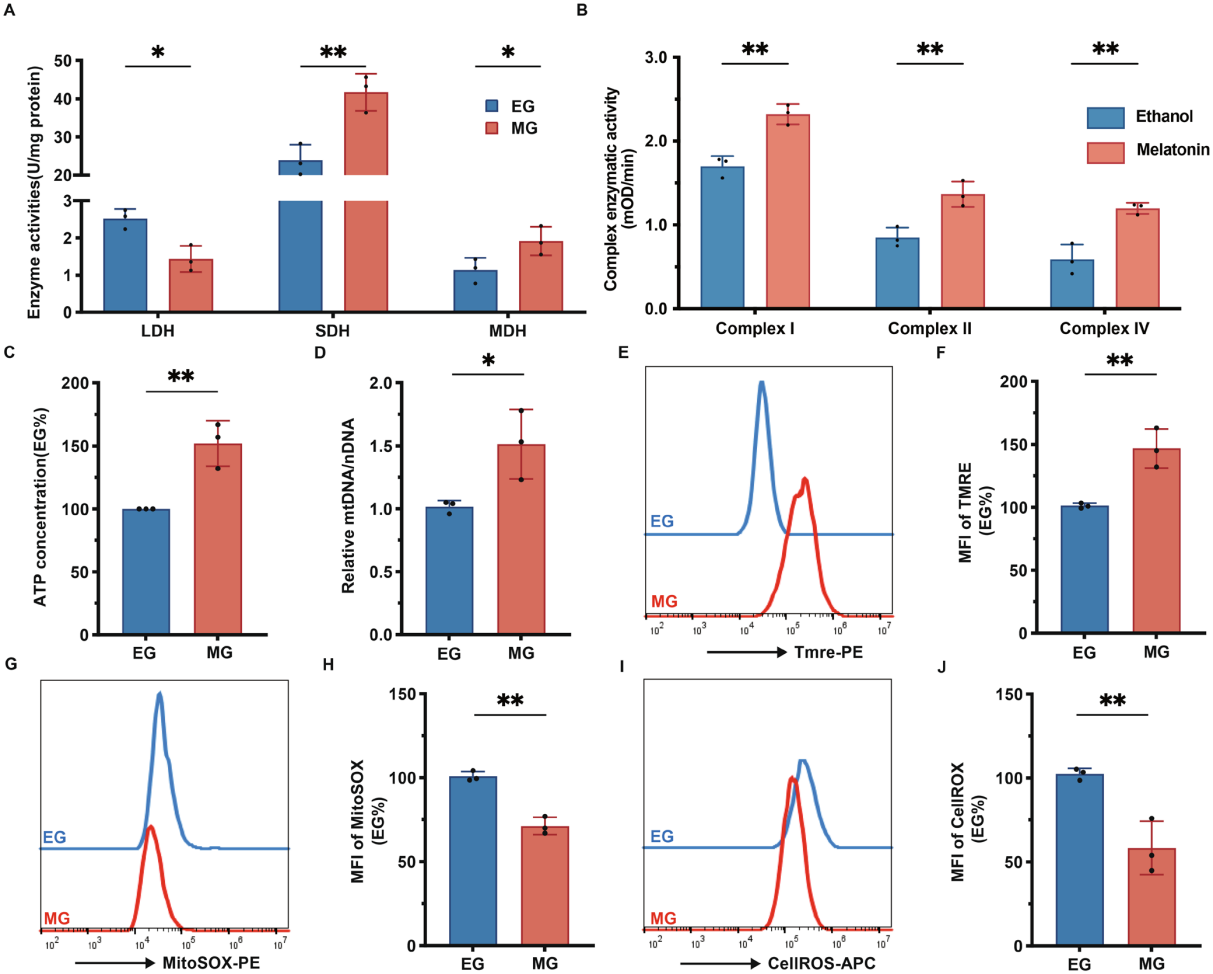
Melatonin enhances the mitochondrial function of muscle fibers. (A) Statistical analysis chart of the changes in LDH, SDH, and MDH enzyme activities in C2C12 muscle tubes of the EG group and the MG group detected by microplate reader. (B) Statistical analysis chart of the changes in the activities of mitochondrial respiratory chain Complexes I, II, and IV detected by microplate reader. (C) Statistical analysis chart of changes in ATP production detected by microplate reader. (D) Statistical analysis chart of changes in mitochondrial mtDNA content in the EG group and the MG group analyzed by real‐time quantitative PCR. (E) Flow cytometry was used to analyze the changes of mitochondrial membrane potential in the EG group and the MG group. (F) Statistical analysis chart of (E). (G) Flow cytometry was used to analyze the changes in mtROS levels in cells of the EG group and the MG group. (H) Statistical analysis chart of (G). (I) Flow cytometry was used to analyze the changes in total ROS levels of cells in the EG group and the MG group. (J) Statistical analysis chart of (I). *p < 0.05, **p < 0.01, compared with the EG group.

As shown in Figure 3A, compared with the EG group, the SDH enzyme activities (*p* < 0.01) and MDH enzyme activities (*p* < 0.05) in the MG group were significantly increased, while the LDH enzyme activity was significantly decreased (*p* < 0.05). As shown in Figure 3B, compared with the EG group, the enzymatic activities of mitochondrial respiratory chain Complexes I, II, and IV in the MG group were significantly increased (*p* < 0.01). As shown in Figure 3C, compared with the EG group, the ATP production in the MG group increased significantly (*p* < 0.01). As shown in Figure 3D, compared with the EG group, the content of mitochondrial mtDNA was significantly increased (*p* < 0.05). As shown in Figure 3E,F, compared with the EG group, the mitochondrial membrane potential in the MG group was significantly increased (*p* < 0.01). As shown in Figure 3E,F, compared with the EG group, the level of mtROS in the MG group cells decreased significantly (*p* < 0.01). As shown in Figure 3I,J, compared with the EG group, the total ROS level of cells in the MG group decreased significantly (*p* < 0.01).

### 3.4 Melatonin Promotes the Activation of the AMPK Signaling Pathway

**FIGURE 4:**
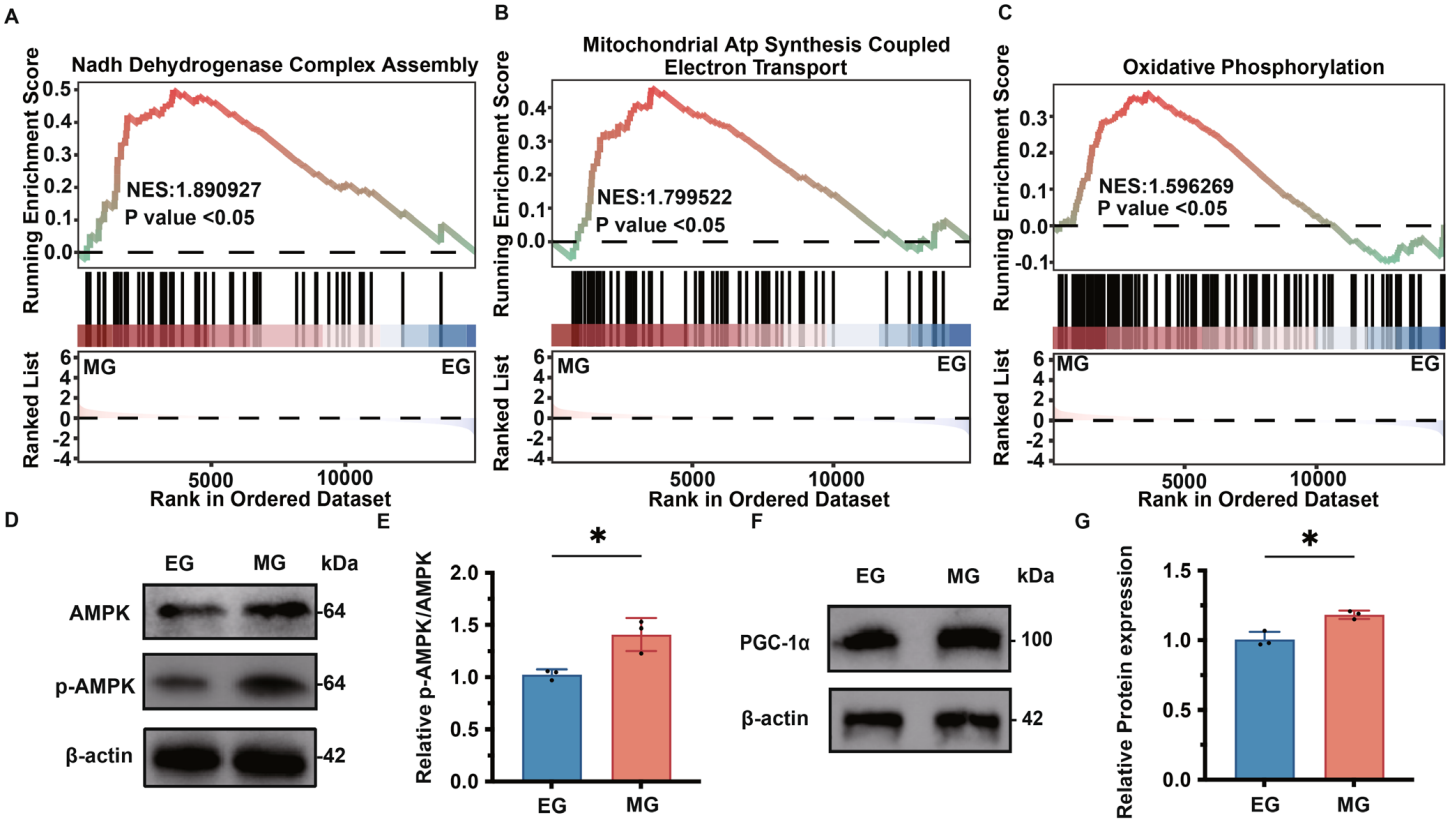
Melatonin promotes the activation of the AMPK signaling pathway. (A) GSEA enrichment analysis was conducted to analyze the enrichment changes of mitochondrial respiratory chain Complex I assembly genes in cells of the MG group and the EG group. (B) GSEA enrichment analysis was conducted on the enrichment changes of mitochondrial ATP synthesis coupled with electron transport genes in the MG group and the EG group. (C) GSEA enrichment analysis of the enrichment changes of oxidative phosphorylation genes in the MG group and the EG group. (D) Western Blot was used to analyze the changes in the protein expression levels of AMPK and p‐AMPK in the MG group and the EG group. (E) Statistical analysis chart of (D). (F) Western Blot was used to analyze the changes in the expression level of PGC‐1α protein in the MG group and the EG group. (G) Statistical analysis chart of (F). *p < 0.05, compared with the EG group.

As illustrated in Figures 4A–D, the MG group exhibited a significant enrichment in genes associated with the assembly of mitochondrial respiratory chain Complex I, mitochondrial ATP synthesis–coupled electron transport, and oxidative phosphorylation, in comparison to the EG group. Figure 4E demonstrates that, relative to the EG group, the MG group showed a decrease in AMPK protein expression levels, whereas the expression levels of phosphorylated AMPK (p‐AMPK) protein increased significantly. This observation was substantiated by statistical analysis (Figure 4F), which revealed that the p‐AMPK/AMPK ratio in the MG group was significantly elevated compared with the EG group (*p* < 0.05). Furthermore, as depicted in Figure 4G,H, the expression level of PGC‐1α protein in the MG group was significantly higher than that in the EG group (*p* < 0.05).

### 3.5 Inhibiting the AMPK Signaling Pathway Weakens the Regeneration of Slow Muscle Fibers Caused by Melatonin

**FIGURE 5:**
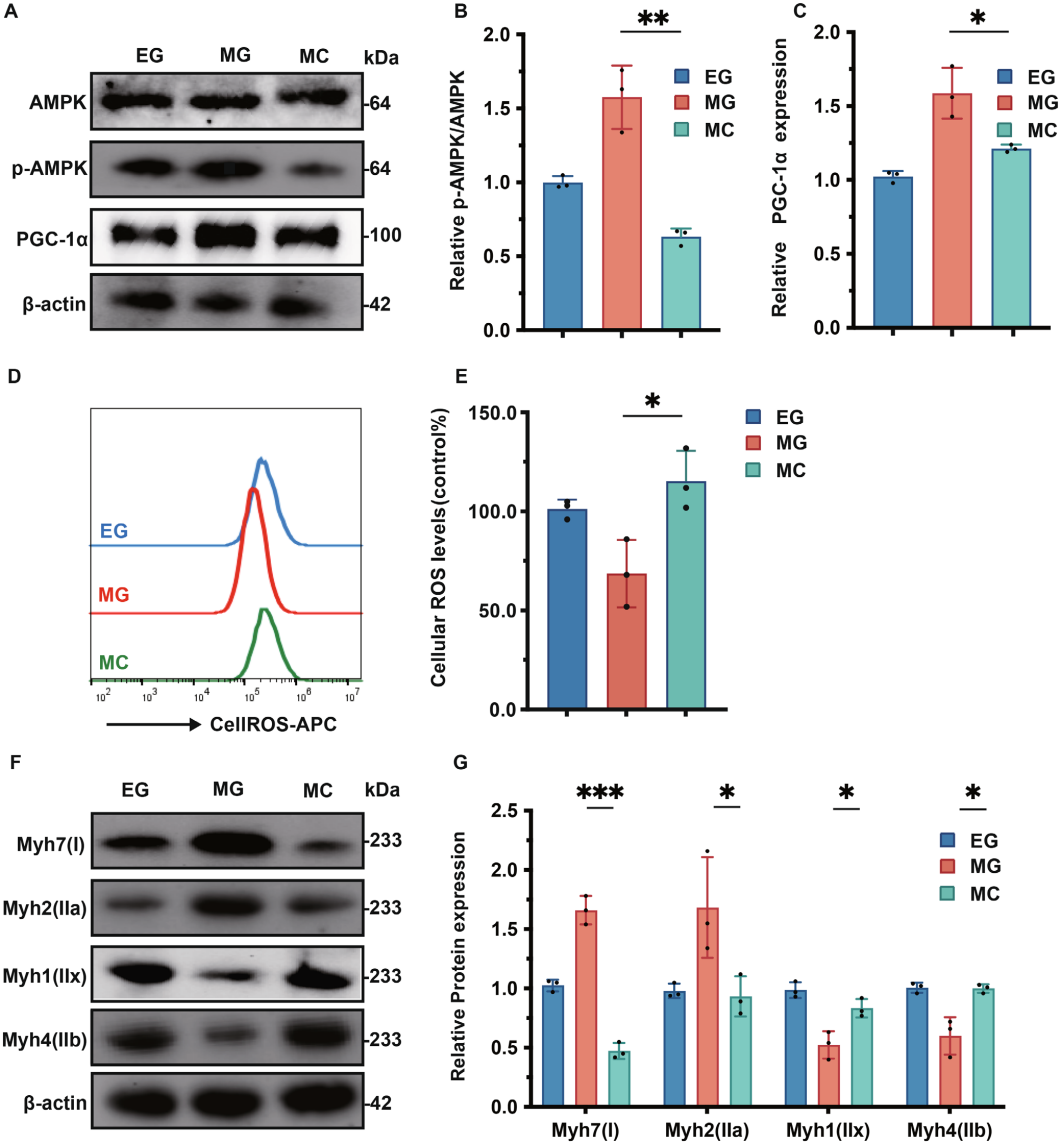
Inhibiting the AMPK signaling pathway weakens the regeneration of slow muscle fibers caused by melatonin. C2C12 cells were cultured in the differentiation medium for 1 day, then pretreated with 50 μM Compound C for 1 h, and finally treated with melatonin (1 μM) for 3 days. (A) Western Blot was used to analyze the changes in the protein expression levels of AMPK, p‐AMPK, and PGC‐1α in cells of the EG group, MG group, and MC group. (B, C) Statistical analysis chart of (A). (D) Flow cytometry was used to analyze the level changes of total ROS in cells of the EG group, MG group, and MC group. (E) Statistical analysis chart of (D). (F) Western Blot was used to analyze the changes in the protein expression levels of Myh7, MhH2, Myh1, and Myh4 in the EG group, MG group, and MC group. (G) Statistical analysis chart of (F). *p < 0.05, **p < 0.01, ***p < 0.001, compared with the MG group.

As illustrated in Figure 5A–C, the MC group exhibited a significant reduction in the p‐AMPK/AMPK ratio (*p* < 0.01) and PGC‐1α expression levels (*p* < 0.05) compared to the MG group. Furthermore, as depicted in Figure 5D,E, the total ROS levels in the MC group were significantly elevated relative to the MG group (*p* < 0.05). Additionally, as shown in Figure 5F,G, the expression levels of Myh7 protein (*p* < 0.001) and Myh2 protein (*p* < 0.05) were significantly diminished in the MC group, whereas the expression levels of Myh1 protein and Myh4 protein were significantly increased (*p* < 0.05) when compared to the MG group.

## 4 Discussion

In this study, we elucidated the role of melatonin in modulating skeletal muscle fiber type transition through AMPK‐mediated signaling pathways during post‐injury repair. Traditionally, melatonin has been recognized for its protective role across various cell types and tissues. Utilizing both in vivo and in vitro models, we conducted a comprehensive investigation into the regulatory role and molecular mechanisms by which melatonin influences the regeneration of slow muscle fibers during skeletal muscle injury repair. Mechanistically, melatonin facilitates a broad enhancement of mitochondrial function by activating the AMPK/PGC‐1α signaling pathway. This activation results in increased activity of respiratory chain Complexes I, II, and IV, augmented ATP synthesis capacity, enhanced mtDNA replication, and a reduction in ROS levels. To ascertain the specificity of this pathway, we employed the AMPK inhibitor Compound C, which effectively inhibited the regulatory impact of melatonin on muscle fiber phenotype. These findings confirm that the AMPK/PGC‐1α pathway is the principal target of melatonin's action, elucidating a novel mechanism by which melatonin induces the regeneration of slow muscle fibers through mitochondrial metabolic pathways.

Muscle fibers are usually classified into type I, type IIa, type IIx, and type IIb fibers according to the MyhC subtype. Type I fibers have a high capillary density and mitochondrial content, thus having a high oxidation capacity but a low glycolytic capacity. Type IIx and type IIb have low capillary density and mitochondrial content, thus having low oxidation capacity and the highest glycolytic capacity. The IIa type metabolic mode is mixed, with medium to high oxidation capacity and relatively high glycolytic capacity. The composition of muscle fiber types is highly malleable and can be changed according to functional requirements, including neuromuscular stimulation, mechanical load, hormones, and aging [6]. Based on the understanding that muscle fiber types are subject to hormonal regulation and exhibit significant plasticity, this study investigated the influence of melatonin on muscle fiber types during the repair of tibialis anterior muscle (TA) injury. In particular, this study investigated the influence of melatonin on the regeneration of slow muscle fibers during the repair process following TA injury, employing both in vivo and in vitro models. Our findings indicate that post‐injury administration of melatonin significantly enhances the regeneration of slow‐twitch muscle fibers (type I/IIa) while inhibiting the formation of fast‐twitch muscle fibers (type IIb). However, given that the in vivo model employed in this study was limited to the tibialis anterior muscle, which predominantly consists of fast‐twitch fibers (approximately 95%), further research is required to determine whether melatonin exerts similar or differing effects on muscles with varying fiber compositions.

Melatonin is mainly produced by the pineal gland of mammals and is the final product of the biosynthetic pathway of tryptophan and serotonin [1]. Melatonin plays a protective role in various cell and tissue types, such as participating in the detoxification of ROS and various free radical intermediates, participating in the expression of antioxidant enzymes, regulating NF‐κB to resist inflammation and the production of ROS induced by inflammation, and protecting mitochondria [17, 28, 29, 30]. In addition, melatonin improves the orientation and maturation of collagen fibers at the wound site, and accelerates angiogenesis to promote wound healing and scar formation [31]. However, at present, in the related research on skeletal muscle injury, the research on the repair effect of melatonin on skeletal muscle injury is very scarce. Wang et al. [32] found that in skeletal muscle reperfusion injury, melatonin may protect microvessels through its free radical scavenging effect: significantly expanding the diameter of small arteries, improving capillary perfusion, and alleviating endothelial dysfunction. Su et al. [33] and Ge et al. [34] found that melatonin can induce the expression of Pax7 through the Wnt signaling pathway, or enhance mitochondrial energy metabolism and activate mitochondrial antioxidant enzymes through the silencing information of SIRT3, playing a key role in the differentiation of C2C12 myoblasts. However, Chen et al. [35] found that melatonin promotes the proliferation of C2C12 myoblasts and inhibits the myogenic differentiation and myotube formation of mouse C2C12 cells and human primary myoblasts by reducing Wnt/β‐catenin signaling, Myomaker, and Myomixer. Stratos et al. [36] found in the model of blunt injury of skeletal muscle that melatonin significantly upregulates the mRNA of specific G protein‐coupled receptors in skeletal muscle, indicating that in addition to indirectly supporting the systemic effect of injured muscle recovery, melatonin can also directly act on the cell population in the injured muscle. Melatonin can also inhibit apoptosis by regulating apoptosis‐related signaling pathways, increase the number of satellite cells, reduce inflammation, and support muscle repair after injury. This study found that in the in vivo model, the number of type I muscle fibers and the expression levels of Myh7 and Myh2 proteins in the FM group were significantly higher than those in the FG group, while the expression levels of Myh1 and Myh4 were significantly lower than those in the FG group. This proved that the injection of melatonin after injury significantly stimulated the regeneration of type I and IIa muscle fibers, while reducing the regeneration of type IIb muscle fibers. Alterations in the proportion of fast to slow muscle fibers within the tibialis anterior muscle suggest that melatonin promotes a differentiation bias towards slow muscle fibers following muscle injury, thereby facilitating muscle repair. More and more studies have proved that at the metabolic level, most differentiated satellite cells turn to oxidative phosphorylation (OXPHOS), which is crucial for terminal myogenic differentiation. Differentiated satellite cells have higher mitochondrial mass, increased levels of mitochondrial respiratory chain complexes, and upregulated tricarboxylic acid cycle enzymes, all of which maintain the high energy requirements of mature muscles [3]. Meanwhile, the reduction of mitochondrial network remodeling has been proven many times to lower the differentiation ability of cultured myoblasts and reduce the regenerative ability of skeletal muscle tissue [37]. Therefore, mitochondria play an important role in the myogenic differentiation of satellite cells and the regeneration of skeletal muscle. In the in vitro model of this study, the mitochondrial mass, mitochondrial mtDNA content, and mitochondrial membrane potential in C2C12 cells of the MG group were significantly higher than those of the EG group, while the mtROS level and total ROS level of the MG group were significantly lower than those of the EG group, indicating that the mitochondrial function in the cells was significantly enhanced, which would lead to an increase in cell oxygen consumption. This is consistent with the result in this study that the OCR curve of the MG group was higher than that of the EG group (both the basal respiratory rate and the maximum respiratory rate increased). In addition, compared with the EG group, the enzyme activities of SDH, MDH, and mitochondrial respiratory chain Complexes I, II, and IV in the MG group were significantly increased, while the enzyme activity of LDH was significantly decreased. This indicates that the metabolic level of C2C12 cells shifted from glycolysis to OXPHOS, and the level of mitochondrial respiratory chain complexes increased, which was consistent with the metabolic changes of activated and differentiated satellite cells. This study also found that the expression levels of Myh7 and Myh2 proteins and their mRNA transcription levels in the MG group were significantly increased, while the expression levels of Myh1 and Myh4 proteins and their mRNA transcription levels were significantly decreased. This indicates that melatonin promotes the differentiation of C2C12 myoblasts towards slow muscle fibers. In conclusion, melatonin can promote the regeneration of slow muscle fibers in the injured tibial anterior muscle, which mainly undergo glycolysis, into slow muscle fibers that mainly undergo oxidative metabolism, enhance mitochondrial function, and adapt to the changes in energy requirements during muscle injury repair, which is beneficial for muscle injury repair.

AMPK is a heterotrimer complex composed of subunits α (α1 and α2), β (β1 and β2), and γ (γ1, γ2, and γ3). The catalytic domain is contained in the α subunit, while the β and γ subunits have regulatory functions [38]. In vitro models, AMPK activation inhibits the proliferation and differentiation of C2C12 myoblasts. However, the deficiency of AMPK in satellite cells within the body can hinder normal muscle regeneration after injury. AMPKα1 is the main catalytic subtype in quiescent, activated, and differentiated satellite cells. When AMPKα1 is knocked down, the regeneration of the damaged muscle is impaired (compared with the wild type), which is related to the reduction in the number of satellite cells and the expression of Pax7, Myf5, and Myogenin [39]. Furthermore, AMPK has specific regulatory effects on various aspects of mitochondrial biology and homeostasis [40]. These aspects include controlling the number of mitochondria by stimulating mitochondrial biogenesis, regulating the shape of the mitochondrial network in cells, and controlling mitochondrial quality by regulating autophagy and mitochondrial autophagy. Mitochondria are organelles in eukaryotes that produce respiratory ATP and are crucial for energy production, cell survival, and stress regulation. Mitochondrial regeneration refers to the process of mitochondrial renewal and repair within cells. It involves de novo mitochondrial synthesis (biogenesis), mitochondrial autophagy, as well as mitochondrial fusion and division, among other steps. Peroxisome proliferator‐activated receptor (PPAR)‐γ coactivator 1α (PGC1α) is an important component of stress‐induced mitochondrial biogenesis and also an important regulator of mitochondrial oxidative capacity. PGC‐1α enriches the mechanisms required for cells and metabolism by interacting with factors such as NRF‐1/2 and PPARγ. For example: AMPK phosphorylation [41] or SIRT1 deacetylation [42] activates PGC‐1α and stimulates the activation of various nuclear transcription factors, such as NRF‐1/2. The expression of NRF‐1/2 induces the expression of TFAM, which is transported to mitochondria to bind to mtDNA and activate transcription and replication. PPARγ controls the proteins involved in mitochondrial biogenesis regulation by regulating the activity of PGC‐1α itself, including promoting the expression of OXPHOS genes in the nucleus and mitochondria, stimulating mtDNA replication, thereby enhancing mitochondrial respiratory function and energy metabolism levels, and reducing the production of ROS [43]. Furthermore, PGC1α can directly bind to the intron regions of subsets of mRNA encoding metabolic genes (including mitochondrial ATP transporter genes) through its RNA binding domain to regulate its gene expression [44]. The results of GSEA enrichment analysis showed that the MG group significantly enriched the genes related to mitochondrial respiratory chain Complex I assembly, mitochondrial ATP synthesis coupled electron transport, and oxidative phosphorylation compared with the EG group. All these genes were directly related to mitochondrial function. The significant enrichment in the MG group indicates that the mitochondrial function of C2C12 cells in the MG group is significantly enhanced. As the central regulator of energy homeostasis, AMPK coordinates metabolic pathways, thereby balancing nutrient supply and energy demand [45]. The mitochondrial function was significantly enhanced, and the energy metabolism level of the cell increased accordingly. As the AMPK pathway is an energy‐sensing pathway, we speculate that the AMPK pathway may be affected by changes in energy metabolism. Through the data analysis of Western Blot in this study, it was found that compared with the EG group, the p‐AMPK/AMPK ratio in the MG group was significantly increased, indicating an improved activation level of AMPK. The function of the AMPK signaling pathway was verified and the MC group was established. The research found that compared with the MG group, the expression levels of Myh7 and Myh2 proteins in the MC group were significantly decreased, while the expression levels of Myh1 and Myh4 proteins were significantly increased, suggesting that the AMPK pathway plays an important role in promoting the regeneration of slow muscle fibers in muscle injury repair. Furthermore, the enhancement of cellular metabolic capacity is related to the remodeling of the mitochondrial network [3]. PGC1α, as an important regulatory factor among them, can be phosphorylated by AMPK to activate other nuclear transcription factors [41]. Through the verification that the expression level of PGC‐1α protein in the MG group was significantly elevated compared to the EG and MC groups, we demonstrated that AMPK facilitates the regeneration of slow muscle fibers post‐injury by modulating its downstream target, PGC‐1α.

This study investigates the effects of melatonin on the regeneration of slow‐twitch muscle fibers during the skeletal muscle injury repair process, alongside its potential molecular mechanisms, utilizing a comprehensive series of in vivo and in vitro experiments. This research offers a novel perspective on the clinical application of melatonin. Our findings indicate that melatonin facilitates the conversion of fast‐twitch muscle fibers into slow‐twitch muscle fibers. Fast‐twitch muscle fibers, categorized as type IIX and type IIB, are characterized by innervation from large motor neurons, limited capillary networks, and well‐developed anaerobic metabolism and sarcoplasmic reticulum, contributing to substantial muscle strength, which is closely associated with physical activity. In contrast, slow‐twitch muscle fibers, primarily type I oxidative fibers, are innervated by small motor neurons and possess extensive capillary networks, robust oxidative and mitochondrial functions, fatigue resistance, and enhanced repair capabilities. Consequently, compared to fast‐twitch fibers, slow‐twitch fibers may provide a more conducive microenvironment for recovery following injury. Notably, the dense capillary networks in slow‐twitch fibers can expedite energy supply and support the reparative processes.

## 5 Conclusion

Following skeletal muscle injury, satellite cells play a crucial role in the regeneration and differentiation of muscle fibers. Notably, melatonin has been shown to influence this differentiation process by promoting the regeneration of slow‐twitch muscle fibers. This effect is ultimately observed as a decrease in fast‐twitch muscle fibers and an increase in slow‐twitch muscle fibers. Mechanistically, melatonin primarily activates the AMPK/PGC‐1α signaling pathway, thereby enhancing mitochondrial function within muscle fibers. This enhancement includes improvements in mitochondrial oxidative respiratory efficiency, the activity of the mitochondrial respiratory chain complexes, mitochondrial mass, ATP, and reactive oxygen species (ROS) production, and membrane potential. Consequently, these changes facilitate the regeneration of slow muscle fibers.

## Author Contributions

W.X.: Conceptualization, methodology, investigation, data curation, writing. Z.H.: Conceptualization, methodology, writing, project administration, writing. Y.X.: Software, validation, data curation. K.H.: Data Curation, visualization, investigation. S.L.: Data Curation, investigation. M.F.: Data curation, investigation. Y.G.: Supervision, project administration, funding acquisition, resources. Z.Z.: Validation, writing – review and editing, supervision, resources. M.Z.: Conceptualization, methodology, validation, investigation, data curation, writing – original draft, writing – review and editing, visualization, supervision, project administration, funding acquisition, resources.

## Conflicts of Interest

The authors declare no conflicts of interest.

## Data Availability

The authors have nothing to report.
